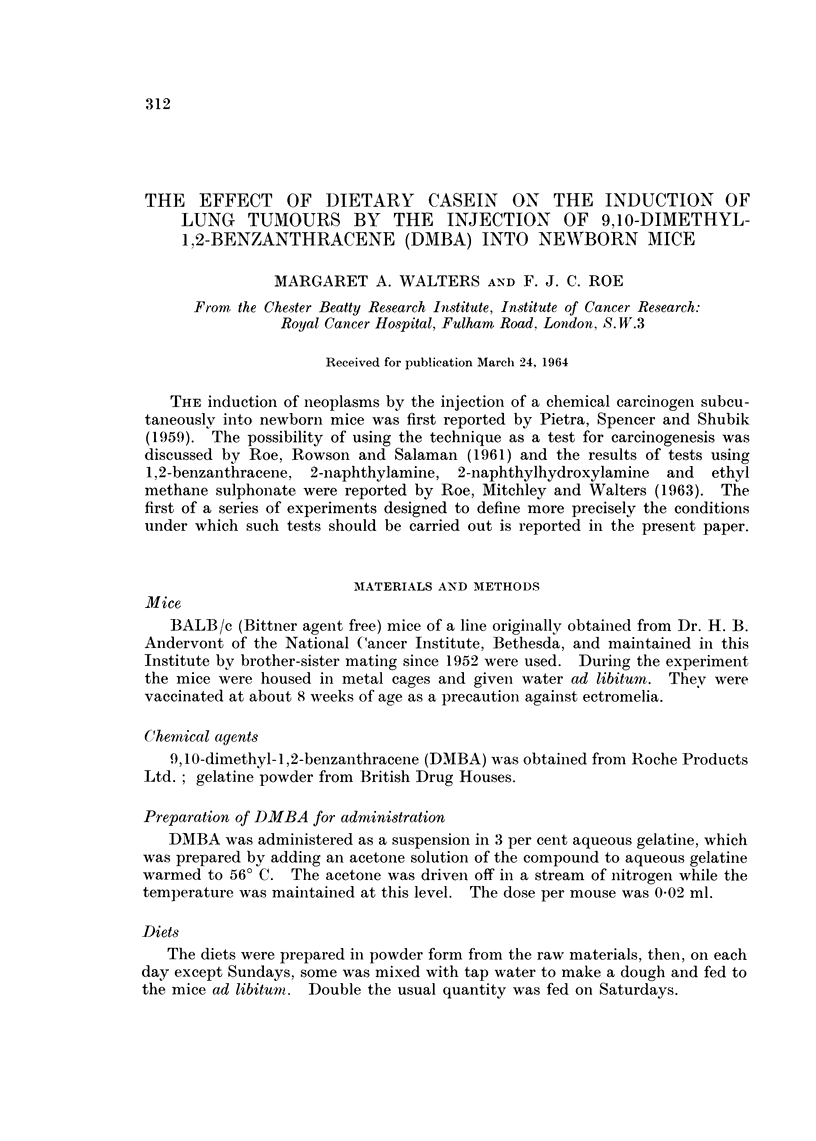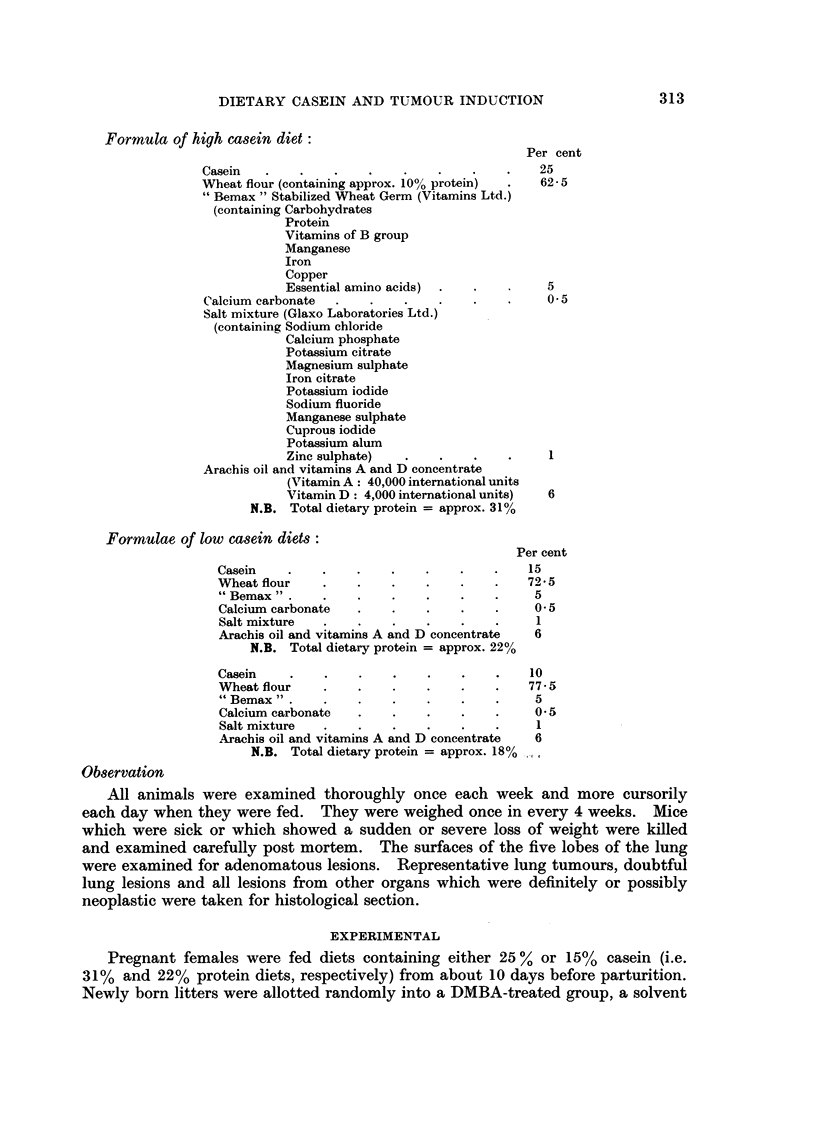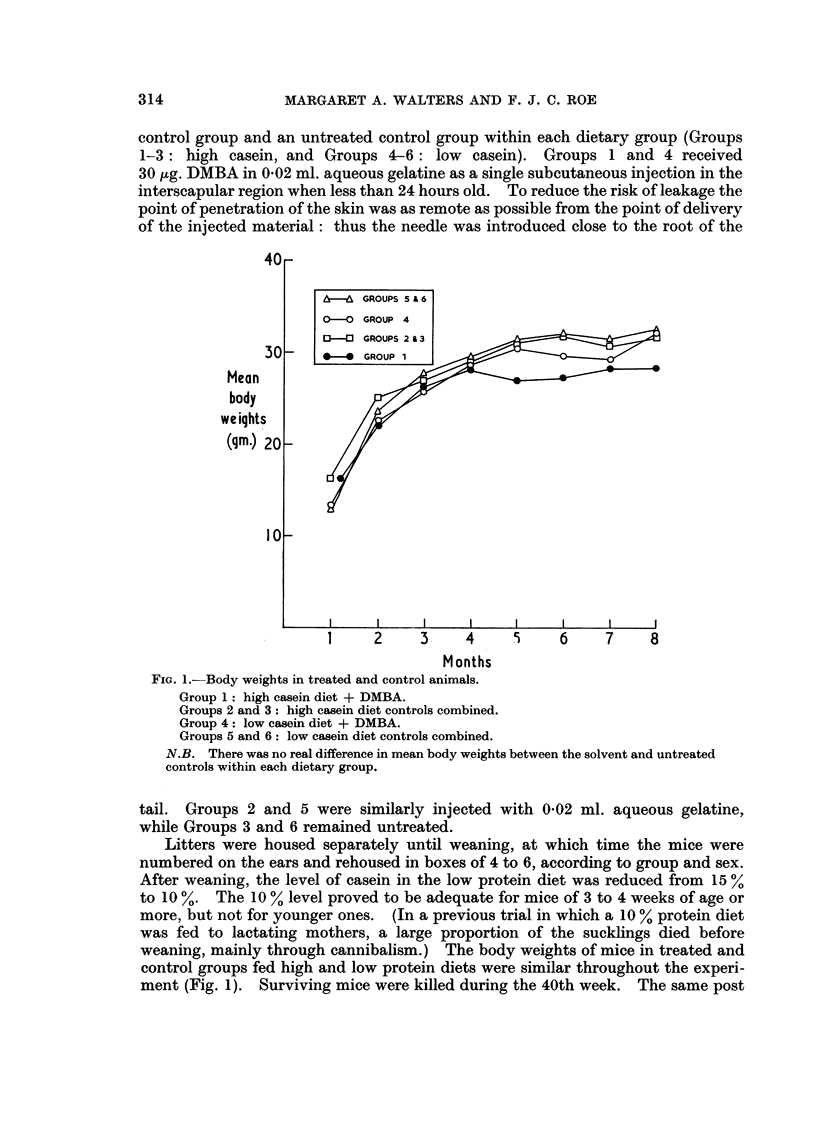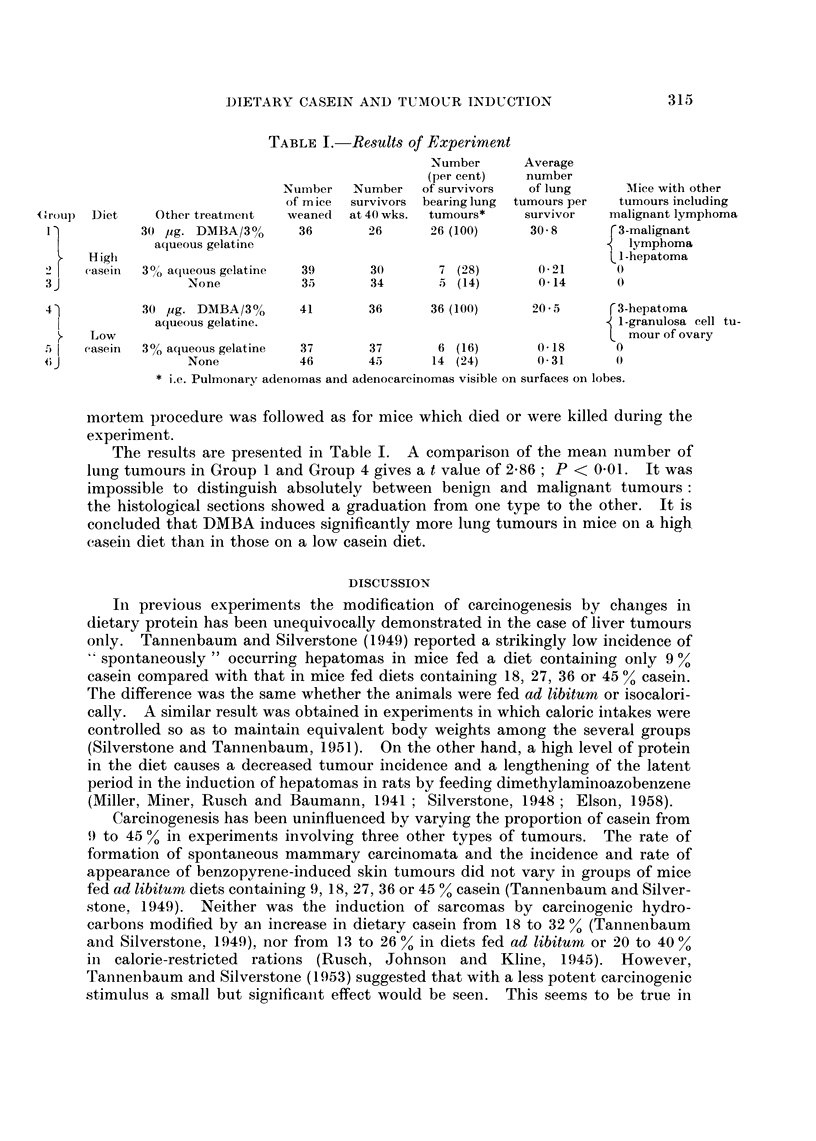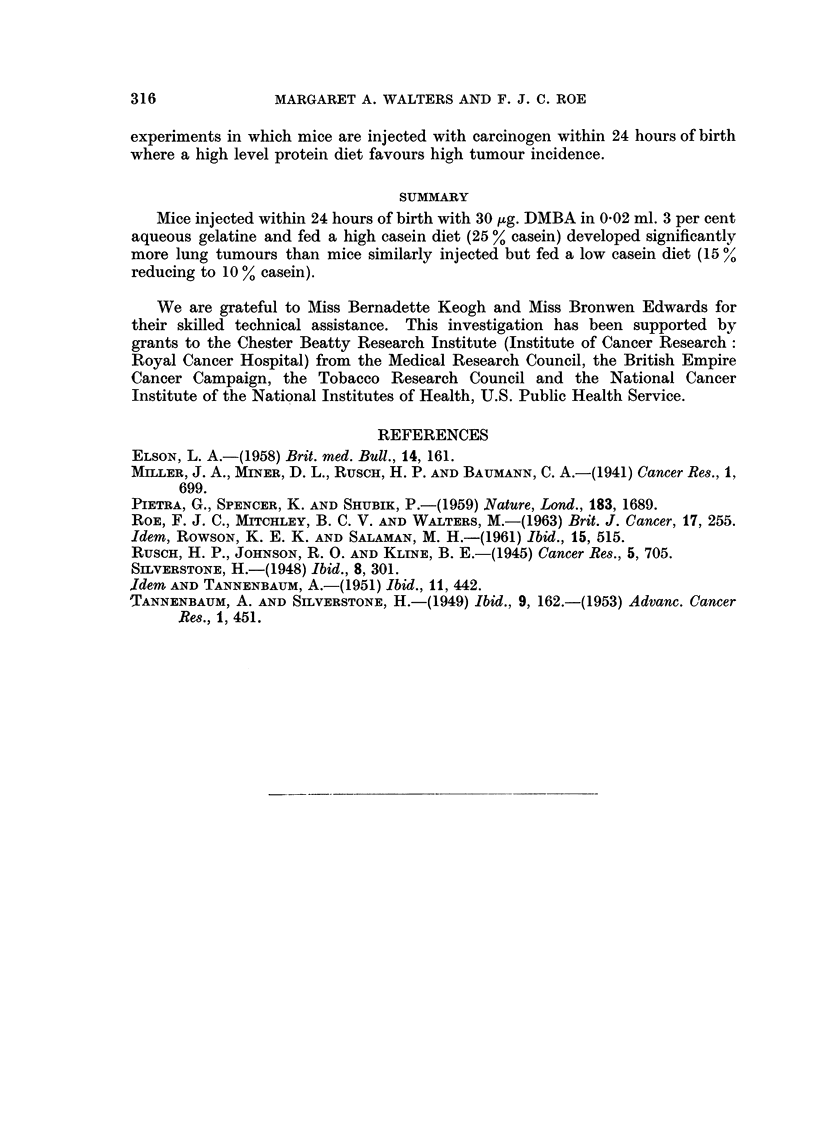# The Effect of Dietary Casein on the Induction of Lung Tumours by the Injection of 9,10-Dimethyl-1,2-Benzanthracene (DMBA) into Newborn Mice

**DOI:** 10.1038/bjc.1964.36

**Published:** 1964-06

**Authors:** Margaret A. Walters, F. J. C. Roe


### THE EFFECT OF DIETARY CASEIN ON THE INDUCTION OF LUNG TUMOURS BY THE INJECTION OF 9,10-DIMETHYL-1,2-BENZANTHRACENE (DMBA) INTO NEWBORN MICE

MARGARET A. WALTERS AND F. J. C. ROE

*From the Chester Beatty Research Institute, Institute of Cancer Research: Royal Cancer Hospital, Fulham Road, London, S.W.3*

Received for publication March 24, 1964

THE induction of neoplasms by the injection of a chemical carcinogen subcutaneously into newborn mice was first reported by Pietra, Spencer and Shubik (1959). The possibility of using the technique as a test for carcinogenesis was discussed by Roe, Rowson and Salaman (1961) and the results of tests using 1,2-benzanthracene, 2-naphthylamine, 2-naphthylhydroxylamine and ethyl methane sulphonate were reported by Roe, Mitchley and Walters (1963). The first of a series of experiments designed to define more precisely the conditions under which such tests should be carried out is reported in the present paper.

### MATERIALS AND METHODS

### *Mice*

BALB/c (Bittner agent free) mice of a line originally obtained from Dr. H. B. Andervont of the National Cancer Institute, Bethesda, and maintained in this Institute by brother-sister mating since 1952 were used. During the experiment the mice were housed in metal cages and given water *ad libitum*. They were vaccinated at about 8 weeks of age as a precaution against ectromelia.

### *Chemical agents*

9,10-dimethyl-1,2-benzanthracene (DMBA) was obtained from Roche Products Ltd. ; gelatine powder from British Drug Houses.

### *Preparation of DMBA for administration*

DMBA was administered as a suspension in 3 per cent aqueous gelatine, which was prepared by adding an acetone solution of the compound to aqueous gelatine warmed to 56° C. The acetone was driven off in a stream of nitrogen while the temperature was maintained at this level. The dose per mouse was 0·02 ml.

### *Diets*

The diets were prepared in powder form from the raw materials, then, on each day except Sundays, some was mixed with tap water to make a dough and fed to the mice *ad libitum*. Double the usual quantity was fed on Saturdays.

*Formula of high casein diet :*

| | Per cent |
|---|---|
| Casein | 25 |
| Wheat flour (containing approx. 10% protein) | 62·5 |
| " Bemax " Stabilized Wheat Germ (Vitamins Ltd.) (containing Carbohydrates, Protein, Vitamins of B group, Manganese, Iron, Copper, Essential amino acids) | 5 |
| Calcium carbonate | 0·5 |
| Salt mixture (Glaxo Laboratories Ltd.) (containing Sodium chloride, Calcium phosphate, Potassium citrate, Magnesium sulphate, Iron citrate, Potassium iodide, Sodium fluoride, Manganese sulphate, Cuprous iodide, Potassium alum, Zinc sulphate) | 1 |
| Arachis oil and vitamins A and D concentrate (Vitamin A : 40,000 international units Vitamin D : 4,000 international units) | 6 |

**N.B.** Total dietary protein = approx. 31%

*Formulae of low casein diets :*

| | Per cent |
|---|---|
| Casein | 15 |
| Wheat flour | 72·5 |
| " Bemax " | 5 |
| Calcium carbonate | 0·5 |
| Salt mixture | 1 |
| Arachis oil and vitamins A and D concentrate | 6 |

**N.B.** Total dietary protein = approx. 22%

| | |
|---|---|
| Casein | 10 |
| Wheat flour | 77·5 |
| " Bemax " | 5 |
| Calcium carbonate | 0·5 |
| Salt mixture | 1 |
| Arachis oil and vitamins A and D concentrate | 6 |

**N.B.** Total dietary protein = approx. 18%

*Observation*

All animals were examined thoroughly once each week and more cursorily each day when they were fed. They were weighed once in every 4 weeks. Mice which were sick or which showed a sudden or severe loss of weight were killed and examined carefully post mortem. The surfaces of the five lobes of the lung were examined for adenomatous lesions. Representative lung tumours, doubtful lung lesions and all lesions from other organs which were definitely or possibly neoplastic were taken for histological section.

### EXPERIMENTAL

Pregnant females were fed diets containing either 25% or 15% casein (i.e. 31% and 22% protein diets, respectively) from about 10 days before parturition. Newly born litters were allotted randomly into a DMBA-treated group, a solvent control group and an untreated control group within each dietary group (Groups 1–3 : high casein, and Groups 4–6 : low casein). Groups 1 and 4 received 30 $\mu$g. DMBA in 0·02 ml. aqueous gelatine as a single subcutaneous injection in the interscapular region when less than 24 hours old. To reduce the risk of leakage the point of penetration of the skin was as remote as possible from the point of delivery of the injected material : thus the needle was introduced close to the root of the

FIG. 1.—Body weights in treated and control animals.
Group 1 : high casein diet + DMBA.
Groups 2 and 3 : high casein diet controls combined.
Group 4 : low casein diet + DMBA.
Groups 5 and 6 : low casein diet controls combined.

*N.B.* There was no real difference in mean body weights between the solvent and untreated controls within each dietary group.

tail. Groups 2 and 5 were similarly injected with 0·02 ml. aqueous gelatine, while Groups 3 and 6 remained untreated.

Litters were housed separately until weaning, at which time the mice were numbered on the ears and rehoused in boxes of 4 to 6, according to group and sex. After weaning, the level of casein in the low protein diet was reduced from 15 % to 10 %. The 10 % level proved to be adequate for mice of 3 to 4 weeks of age or more, but not for younger ones. (In a previous trial in which a 10 % protein diet was fed to lactating mothers, a large proportion of the sucklings died before weaning, mainly through cannibalism.) The body weights of mice in treated and control groups fed high and low protein diets were similar throughout the experiment (Fig. 1). Surviving mice were killed during the 40th week. The same post

TABLE I.—*Results of Experiment*

| Group | Diet | Other treatment | Number of mice weaned | Number survivors at 40 wks. | Number (per cent) of survivors bearing lung tumours* | Average number of lung tumours per survivor | Mice with other tumours including malignant lymphoma |
|---|---|---|---|---|---|---|---|
| 1 | High casein | 30 $\mu$g. DMBA/3% aqueous gelatine | 36 | 26 | 26 (100) | 30·8 | 3-malignant lymphoma; 1-hepatoma |
| 2 | High casein | 3% aqueous gelatine | 39 | 30 | 7 (28) | 0·21 | 0 |
| 3 | High casein | None | 35 | 34 | 5 (14) | 0·14 | 0 |
| 4 | Low casein | 30 $\mu$g. DMBA/3% aqueous gelatine. | 41 | 36 | 36 (100) | 20·5 | 3-hepatoma; 1-granulosa cell tumour of ovary |
| 5 | Low casein | 3% aqueous gelatine | 37 | 37 | 6 (16) | 0·18 | 0 |
| 6 | Low casein | None | 46 | 45 | 14 (24) | 0·31 | 0 |

* i.e. Pulmonary adenomas and adenocarcinomas visible on surfaces on lobes.

mortem procedure was followed as for mice which died or were killed during the experiment.

The results are presented in Table I. A comparison of the mean number of lung tumours in Group 1 and Group 4 gives a $t$ value of 2·86 ; $P < 0{\cdot}01$. It was impossible to distinguish absolutely between benign and malignant tumours : the histological sections showed a graduation from one type to the other. It is concluded that DMBA induces significantly more lung tumours in mice on a high casein diet than in those on a low casein diet.

### DISCUSSION

In previous experiments the modification of carcinogenesis by changes in dietary protein has been unequivocally demonstrated in the case of liver tumours only. Tannenbaum and Silverstone (1949) reported a strikingly low incidence of " spontaneously " occurring hepatomas in mice fed a diet containing only 9 % casein compared with that in mice fed diets containing 18, 27, 36 or 45 % casein. The difference was the same whether the animals were fed *ad libitum* or isocalorically. A similar result was obtained in experiments in which caloric intakes were controlled so as to maintain equivalent body weights among the several groups (Silverstone and Tannenbaum, 1951). On the other hand, a high level of protein in the diet causes a decreased tumour incidence and a lengthening of the latent period in the induction of hepatomas in rats by feeding dimethylaminoazobenzene (Miller, Miner, Rusch and Baumann, 1941 ; Silverstone, 1948 ; Elson, 1958).

Carcinogenesis has been uninfluenced by varying the proportion of casein from 9 to 45 % in experiments involving three other types of tumours. The rate of formation of spontaneous mammary carcinomata and the incidence and rate of appearance of benzopyrene-induced skin tumours did not vary in groups of mice fed *ad libitum* diets containing 9, 18, 27, 36 or 45 % casein (Tannenbaum and Silverstone, 1949). Neither was the induction of sarcomas by carcinogenic hydrocarbons modified by an increase in dietary casein from 18 to 32 % (Tannenbaum and Silverstone, 1949), nor from 13 to 26 % in diets fed *ad libitum* or 20 to 40 % in calorie-restricted rations (Rusch, Johnson and Kline, 1945). However, Tannenbaum and Silverstone (1953) suggested that with a less potent carcinogenic stimulus a small but significant effect would be seen. This seems to be true in experiments in which mice are injected with carcinogen within 24 hours of birth where a high level protein diet favours high tumour incidence.

SUMMARY

Mice injected within 24 hours of birth with 30 μg. DMBA in 0·02 ml. 3 per cent aqueous gelatine and fed a high casein diet (25 % casein) developed significantly more lung tumours than mice similarly injected but fed a low casein diet (15 % reducing to 10 % casein).

We are grateful to Miss Bernadette Keogh and Miss Bronwen Edwards for their skilled technical assistance. This investigation has been supported by grants to the Chester Beatty Research Institute (Institute of Cancer Research : Royal Cancer Hospital) from the Medical Research Council, the British Empire Cancer Campaign, the Tobacco Research Council and the National Cancer Institute of the National Institutes of Health, U.S. Public Health Service.

### REFERENCES

ELSON, L. A.—(1958) *Brit. med. Bull.*, **14**, 161.
MILLER, J. A., MINER, D. L., RUSCH, H. P. AND BAUMANN, C. A.—(1941) *Cancer Res.*, **1**, 699.
PIETRA, G., SPENCER, K. AND SHUBIK, P.—(1959) *Nature, Lond.*, **183**, 1689.
ROE, F. J. C., MITCHLEY, B. C. V. AND WALTERS, M.—(1963) *Brit. J. Cancer*, **17**, 255.
*Idem*, ROWSON, K. E. K. AND SALAMAN, M. H.—(1961) *Ibid.*, **15**, 515.
RUSCH, H. P., JOHNSON, R. O. AND KLINE, B. E.—(1945) *Cancer Res.*, **5**, 705.
SILVERSTONE, H.—(1948) *Ibid.*, **8**, 301.
*Idem* AND TANNENBAUM, A.—(1951) *Ibid.*, **11**, 442.
TANNENBAUM, A. AND SILVERSTONE, H.—(1949) *Ibid.*, **9**, 162.—(1953) *Advanc. Cancer Res.*, **1**, 451.